# Free will and neurosurgical resections of the supplementary motor area: a critical review

**DOI:** 10.1007/s00701-021-04748-9

**Published:** 2021-02-10

**Authors:** Rickard L Sjöberg

**Affiliations:** 1 Department of Clinical Science, Umeå University, Umeå, Sweden; 2 Department of Clinical Science, Neurosciences, Umeå University, S901 85 Umeå, Sweden

**Keywords:** Free will, Glioma, SMA syndrome, Executive function, Cognitive control, Consciousness

## Abstract

**Background:**

Research suggests that unconscious activity in the supplementary motor area (SMA) precedes not only certain simple motor actions but also the point at which we become aware of our intention to perform such actions. The extent to which these findings have implications for our understanding of the concepts of free will and personal responsibility has been subject of intense debate during the latest four decades.

**Methods:**

This research is discussed in relation to effects of neurosurgical removal of the SMA in a narrative review.

**Results:**

Removal of the SMA typically causes a transient inability to perform non-stimulus-driven, voluntary actions. This condition, known as the SMA syndrome, does not appear to be associated with a loss of sense of volition but with a profound disruption of executive function/cognitive control.

**Conclusions:**

The role of the SMA may be to serve as a gateway between the corticospinal tract and systems for executive function. Such systems are typically seen as tools for conscious decisions. What is known about effects of SMA resections would thus seem to suggest a view that is compatible with concepts of personal responsibility. However, the philosophical question whether free will exists cannot be definitely resolved on the basis of these observations.

Intuitively, many of us tend to believe in what is often described as the ideo-motor theory of behavior. According to this view, at least some of our actions are governed by visions of future outcomes that we have decided to pursue [21]. The popular belief that the proximal cause of human behavior are conscious choices and decisions for which we can be held responsible is broadly consistent with views that have dominated theological and scholarly thinking about the human condition for thousands of years. So, for instance, according to the ancient legend of the garden of Eden, the first humans were expelled from an Earthly paradise as a consequence of a choice to break with a command made by God. This legend, which features prominently both in the Old Testament and the Qur’an, defines the human ability for free choice and the capacity for both good and evil that follow as a consequence as a central theological problem [2, 15].

However, the idea that some human actions are governed by free will is not as evidently valid and straightforward as one might intuitively assume. Some early philosophers such as Lucretius [34] and Aristotle [1] had considerable difficulties in reconciling the existence of free will with their view of nature as governed by chains of causes and effects. Since then, philosophers, scientists, and theologians have continued to struggle with issues such as whether the concept of free will is compatible with our understanding of the deterministic laws of nature and/or compatible with the belief in an almighty God [38].

During the latest decades of the twentieth century, this theological/philosophical debate took an unexpected turn, as hopes were raised that neuroscience was on the verge of providing definitive, empirically based resolutions of these issues. The findings that inspired these hopes suggested that unconscious neural activity in a brain region that can anatomically be referred to as the dorsal medial frontal cortex (dMFC) or functionally, and more specifically, as the supplementary (and pre-supplementary) motor areas (SMA) preceded both voluntary action but also the feeling of deciding to perform the action. Observations that appeared to point in this direction were made both in experiments registering activity in this area and in studies where such activity was induced by direct cortical stimulation. The timeline invited the interpretation that dMFC/SMA activity was the cause of the feeling of wanting and deciding to act [43]. A fairly typical example of this reasoning is given by Wegner [57]:

It seems that conscious wanting is not the beginning of the process of making voluntary movement but rather is one of the events in a cascade that eventually yields such movement. The position of conscious will in the time line suggests perhaps that the experience of will is a link in a causal chain leading to action, but in fact it might not even be that. It might just be a loose end—one of those things, like the action, that is caused by prior brain and mental events.

During the latest 3–4 decades, and in the wake of these initial findings, a host of research has continued to explore the neurobiological basis of human volition and decision-making [10, 11, 14, 16, 32, 39, 42]. However, considering the initial findings regarding SMA function is still, as noted by neurobiologist Robert Sapolsky [44], “virtually ordained [in] any discussion of volition and biology.”

## Focus of the present review

The present review concerns itself mainly with one particular neurosurgical dimension of the neuro-philosophical debate surrounding the relation between dMFC/SMA and conscious free will. When the key findings on this issue were made between 1983 and 1991 [11, 33], experiences from SMA resections in humans had only been reported for approximately six cases [27, 40]. However, today, such resections have become relatively common. This makes it possible to examine experiences from SMA resections in relation to current neuro-philosophical theories on free will. The main purpose of the present narrative review is thus to critically discuss this issue.

## Supplementary motor area function and free will

**Fig. 1:**
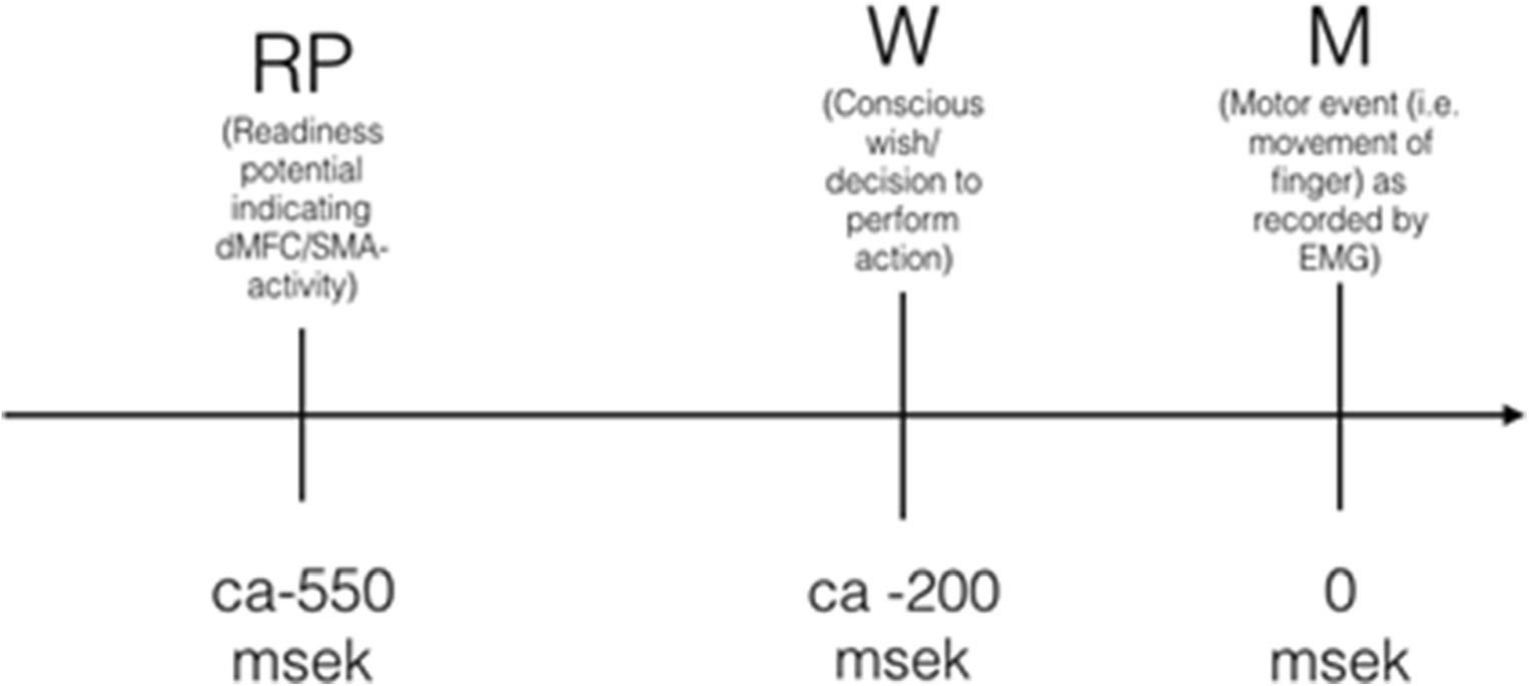
Overview of the main results of the so called Libet experiment

Voluntary, self-initiated movement often appears to be preceded by an electrical potential that can be most clearly observed by electroencephaloeram (EEG) registration over the dMFC/SMA area [25]. In a 1983 experiment by Benjamin Libet et al. [33] that has come to be known as “the Libet experiment,” the exact time at which this “readiness potential” (RP) occurred in relation to a motor movement in the form of a flick of the wrist performed by participants (M) was recorded. In addition, the participants were instructed to choose the exact time at which to perform this action and to report the exact time at which they decided that they wanted (W) to do so. In order to help the participants report the time of their decisions as accurately as possible, they got to watch a dot moving in a circle on a screen with a frequency of one full lap every 2.56 s and were asked to indicate the position of the dot when the decision was made. Activity in the SMA preceded the subjective experience of making the decision to perform the action with roughly 300 ms (Fig. 1). This was the finding that initiated the debate on neuroscience and free will, and the general timeline demonstrated in the original experiment has later been replicated using several different techniques including functional magnetic imaging and single cell recordings from depth electrodes [12, 28].

A second seminal finding regarding the SMA and sense of volition was reported in 1991 by Fried et al. [8], where the results of direct cortical stimulation performed as part of epilepsy surgery evaluations were presented. Here the authors reported that low stimulation intensities over the SMA cause the patient to experience a subjective urge to move contralateral body parts. If the stimulation parameters are more intense, movement would typically be initiated. This movement would be perceived by the patient as voluntary. To many, these observations seemed to provide further support for the notion that SMA activity, even if artificially created, was a direct cause of an illusion that our decisions are based on free unconstrained will.

**Fig. 2:**
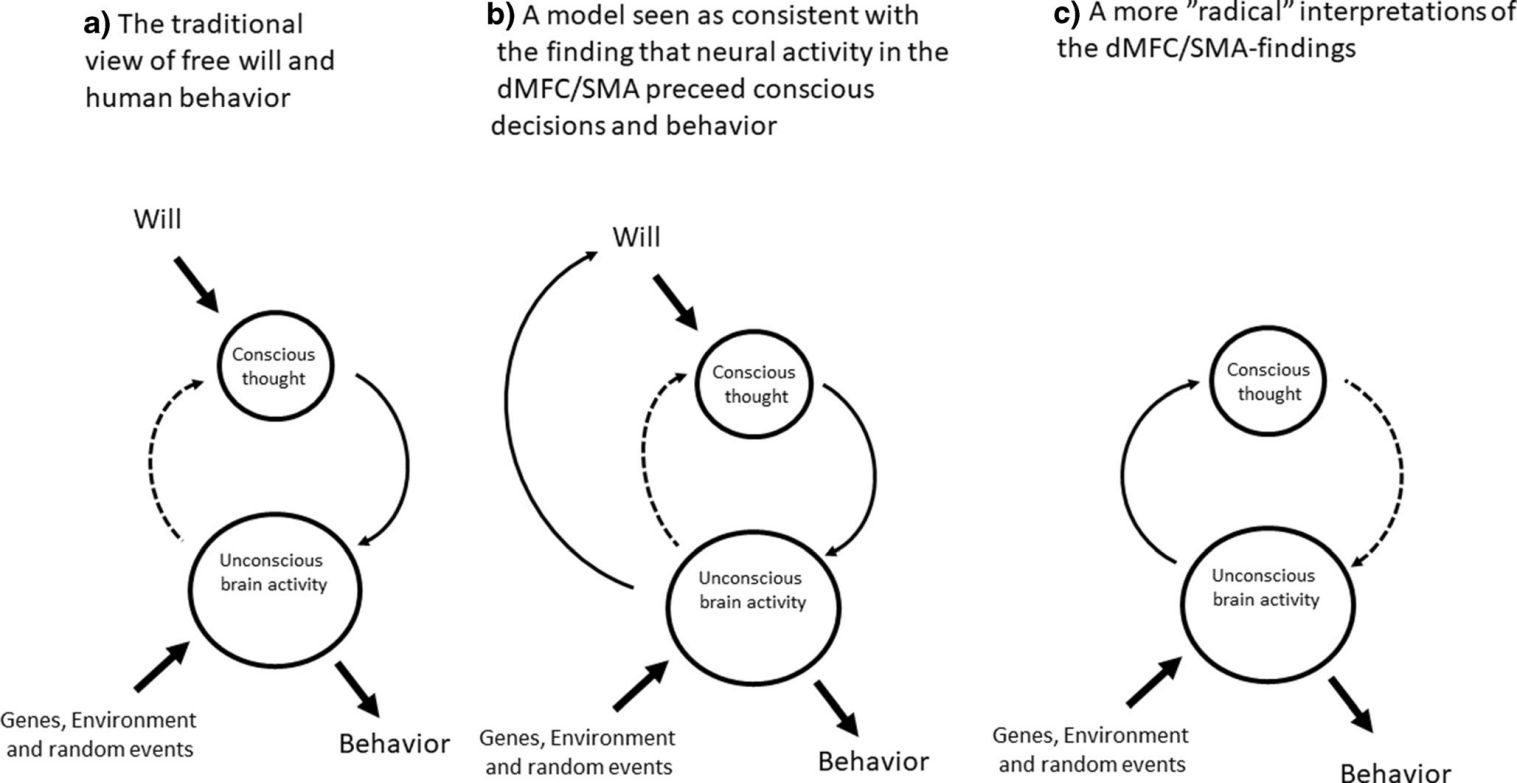
Three models of human behavior in which free will plays different roles. The models B and C are inspired by hypotheses about unconscious physiological activity in the SMA

The main reason that these findings have continued to generate such intense cross-disciplinary interest to this day is of course the potential implications for the attribution of blame and personal responsibility for self-initiated actions. That is, most people appear to hold the opinion that in order for a person to be accountable for an action, such as punching another person in the face, the individual performing the action must have made a conscious decision to do so and be aware of what he or she is doing [45]. What the results of the Libet experiment seems to imply is that self-initiated actions start before we become consciously aware of them. If we accept this pattern as a general model for consequential decision-making, it would appear that no one could ever be held responsible for initiating any actions whatsoever. The consequences of this interpretation of Libet’s findings are illustrated by Cashmore who compares three theoretical models for human behavior (Fig. 2). According to the two latter of these models, unconscious SMA activity is seen as a cause of the experience of wanting and deciding to perform the action but it is also seen as a cause of the action itself, either in a direct (model C in Fig. 2) or indirect fashion (model B in Fig. 2).

## The nature of SMA activity prior to self-initiated movement

As described above, stimulation of the SMA, above a certain threshold of intensity, can induce simple movement in the contralateral part of the body that is often perceived as more or less voluntary. Stimulation on lower parameters appears to induce sensations that have been described as an “urge to move” [11]. Others have reported eliciting the response of the patient feeling as moving without moving [41].

Results of inducing urges to move, or a feeling of moving, have more lately also been described for stimulation of parietal cortex [9]. In this area, increased stimulation did not elicit movement whereas cortical stimulation of the premotor cortex elicited movement that patients were unaware of. Furthermore, fMRI studies suggest that the kind of activity that is associated with the readiness potential in the SMA is preceded by several seconds by activity in other brain regions, such as the frontopolar, parietal, and singular cortices [28, 29, 52].

Simulations using patterns created by a leaky stochastic accumulator appears to create the exact same patterns as the readiness potential [46]. Taken together, this seems to suggest that the SMA serves as a gateway through which other areas of the brain initiates non-stimulus-driven action by causing SMA activity above a certain threshold. Regarding the issue whether the readiness potential precedes only consequential or only non-consequential actions, evidence seems to be conflicting. Report of an absence of the readiness potential for consequential decisions about donating money has been reported [35]. However, others have noted that the readiness potential precedes the decision to jump during bungy jumping [36].

## The ecological validity of the Libet experiment as a model for human decision-making

An important line of discussion and critique of the Libet experiment concerns the question of its ecological validity for the understanding of consequential decision-making [30, 47]. The kinds of decisions studied by Libet et al. concern simple goal-directed motor movements that may be seen as pretty similar to those many of us perform, for instance, in order to get to our workplace in time (staying on track, while putting one foot in front of the other, getting in and out of the car, riding a bike, paying the bus fare, etc.). Such movements, and actions, taking us closer to set future goals and subgoals, are not always monitored in detail by consciousness [3, 13, 18]. If someone would stop us on the way to the morning meeting, pointing out to us the fact that we just recently put our left foot in front of the right one while walking down the corridor, asking us if that particular movement was voluntary, we would probably answer in the affirmative. But if this interviewer would go on and ask us to pinpoint the exact time at which we made the decision to perform the movement, many of us might go further back than 200 ms before the movement. “When I woke up this morning and decided not to call in sick,” might be one possible reply. “When I decided to pursue a neurosurgery residency despite knowing about the long on-call hours” might be another. The point is that having made the decision of which goal to pursue, going through the details such as putting one foot in front or the other, or flicking a wrist at a particular time, is reduced to execution, governed by a system for cognitive control. The mind will go on to monitor these actions to ascertain their adequacy. However, the kind of conscious careful deliberation and weighing of pros and cons that is typically associated with concepts such as “conscious free will” may not be in play for every motor action involved in every step you take.

The neuropsychological concept of executive function/cognitive control is a well-established, albeit somewhat elusive, concept within the field of scientific, cognitive psychology. Lezak et al. [31] define it as involving volition, planning, purposeful action, and effective performance. Braem et al. [7] define it as a set of “higher order processes that are thought to direct, correct, and redirect behavior in line with internal goals and current context.” What is important for our purposes is that theories of executive function implicitly presupposes and rests on an assumption of the existence of consequential conscious decision-making.

If a system for executive function is what induces the readiness potential, SMA activity is useless as an argument against the existence of free will. But if the readiness potential signifies a process during which physiological and biochemical brain processes make consequential selections of future goals for us independently and beyond the reach of conscious awareness, free will may be in jeopardy. If the former hypothesis is right, human intuition seems to be fairly to the point, and William James’ [21] ideo-motor theory may be fairly accurate. If the latter assumption is right, then humankind may have to thoroughly re-evaluate its perception of itself and society, including a thorough re-evaluation of the foundations for the criminal justice system.

## Resections of the SMA and the SMA syndrome

The most important early modern functional description of the SMA was presented by Penfield and Welch [41], who described experiences of direct cortical stimulation of dMFC in a series of 24 patients in addition to effects of unilateral resection of the same area in two monkeys. A fairly consistent finding here is that cortical stimulation elicits vocalization, head turning, and movements. Penfield and Jasper [40] later briefly presented experiences from three patients undergoing resections in the SMA, all of which are described as postoperatively having developed a slowing of movement of extremities and a grasping reflex in the contralateral hand (similar to what Penfield and Welch [41] observed in monkeys). Eventually, however, most of the effects disappeared and only the inability to perform rapidly alternating movements between extremities remained.

More recently, several case series of patients undergoing surgical resection of this area, either as a treatment of gliomas or as part of epilepsy surgery, have been presented [5, 24, 51, 54, 58]. Clinically, these patients are typically described as undergoing a postoperative evolution of a series of symptoms, most of which will eventually resolve, i.e., the so called SMA syndrome. As described by Laplane et al. [27] in a series of 3 patients, the first stage of the syndrome is characterized by a brief initial period of akinetic mutism, which appears to be prolonged in patients with bilateral SMA damage [17]. The second stage involves recovery of function but with persisting reduction of spontaneous motor movement and speech. The last stage is full recovery, with only remaining difficulties in performing rapidly, alternating movements of the hand remaining. Zentner et al. [58], presenting experiences of surgery in 28 patients, describes the mean time to stage three (i.e., resolution of all clinically significant symptoms) as 11 days after surgery, although some patients were affected up to 3 months after the procedure.

The exact neurophysiological mechanisms by which resolution of symptoms takes place are not fully understood. However, case series presenting pre- and postoperative functional magnetic resonance imaging of patients undergoing surgical resections in this area suggests reorganization of functional networks allowing compensation by the intact contralateral SMA [26, 55].

## The SMA syndrome—loss of free will or loss of executive function?

When discussing the implications of the SMA syndrome for the relationship between this brain region and free will, it is important to remember that the central issue here is not whether SMA activity, for instance in the form of a readiness potential, precedes the execution of moments. The central finding is that this activity also precedes what subjects and patients describe as the sensation of becoming conscious of wanting and deciding to perform the action. One interpretation of this finding might be that the sensation of wanting and deciding to perform a given simple motor action at a certain time signifies a continuous self-monitoring [28, 29] performed by a system for executive function in order to ascertain that the individual keeps working in concordance with goals set during previous conscious decisions and choices. The other would be that a center in the SMA actually deceives the conscious self into experiencing a sense of wanting to perform an action that the SMA has already initiated.

If the former interpretation is accurate, a resection of the SMA could be expected to have profound effects on executive function, particularly with regard to the ability to voluntarily initiate speech and contralateral motor actions. If the latter would be true, we would expect profound effects primarily on the ability to make strategic and important decisions and on the ability to “want” to perform certain actions.

Based on the literature and neurosurgical experience, the features of the SMA syndrome appear to be consistent mainly with the first of these two hypotheses. That is, the inability of the individual to use systems for cognitive control for the executions of movement and speech during the SMA syndrome appears to be the central hallmark of the condition. Furthermore, reports of the kind of profound apathy that a dramatic loss of will power should reasonably produce are conspicuously absent from the neurosurgical literature in which SMA resections are described.

More recently, the state of the subjective sense of volition associated with the SMA syndrome was investigated by Stålnacke et al. [44] in a series of 9 brain tumor patients. These patients all experienced different extents of the SMA syndrome either after glioma surgery (8 patients) or resection of a meningioma (1 patient) in the left hemisphere. Symptoms ranged in severity from a period of akinetic mutism in one patient to discrete slowing of speech. The patients were all explicitly asked whether they felt that their impairments during the syndrome were caused by a lack of volition. They were also asked to grade their effort to comply with commands during the neurological examinations as compared with the right side. Five of the patients answered these questions while they were under the influence of the SMA syndrome and 4 answered questions retrospectively.

Results were clear. In their replies, all patients denied feeling a lack of a sense of volition as a reason for their difficulties, and all estimated that they made a 100% effort to comply with commands for all parts of the exam for both affected and unaffected functions. Of course, self-reports such as these, particularly those based on retrospective memories, are subjective interpretations and constructions of reality that can potentially be influenced by a host of cognitive and social factors [50, 56]. Still, despite their limitations, self-reports are also the most direct way to study the nature of such subjective reconstructions of reality. Furthermore, the fact that all answers given by the patients studied by Stålnacke et al. were identical whereas there was variation with regard to at what time the questions were asked in relation to the SMA syndrome may be taken to suggest that the answers convey a subjective experience that is relatively stable across conditions among patients.

In another recent study using an overlapping set of patients, Sjöberg et al. [51] investigated effects of resections in the SMA on known psychological tests for executive function/cognitive control. The first of three tests used was the Color-Word interference test [53] that measures the ability of the individual to describe the color of the letters used to write a word signifying a color (i.e., the word RED written in green). This test taxes the ability of the individual to focus his or her attention on the color and not the word. The second test (the forced left condition of the Bergen dichotic listening task) investigated the ability of the individual to counter the spontaneous tendency of right-handed individuals to prioritize sound heard in the right ear relative to the left [19, 20]. Finally, the relation between the ability to produce words starting with a certain letter relative to the ability to produce words belonging to a certain category was tested. These tests, including some control conditions, were performed prior to surgery, immediately after surgery while subjects were influenced by the SMA syndrome and finally approximately 3 months after surgery. Results showed that while patients were influenced by the SMA syndrome, all measures of executive function/cognitive control were profoundly affected whereas effects on control conditions (i.e., the non-forced condition of the dichotic listening task) were very modest. At 3-month follow-up, after the resolution of the SMA syndrome, effects had disappeared.

Sjöberg et al. did not explicitly discuss the implications of their findings in relation to the neuro-philosophical discussion of the role of SMA resections for the experience of free will. However, taken together with the findings by Stålnacke et al., these results must be seen as giving clear support to the notion that SMA activity may be important in channeling commands from systems for executive function/cognitive control regarding more or less complex pre-planned behaviors. The results also run counter to the notion that SMA activity is central for the subjective experience of free will.

## The reconstructive nature of the monitoring of volition

As has already briefly been discussed, one of the most fundamental principles of cognitive science is that self-reports describing what we see, perceive, remember, feel, or do are fundamentally reconstructive and interpretive. This often serves us well, but there are also examples of situations where illusions, misconceptions, and misunderstandings follow. Examples of such instances range from dramatic events such as the early modern European witch persecutions to more mundane cognitive and perceptual illusions that are commonplace in introductory textbooks of cognitive psychology [6, 21–23, 37, 48, 49]. The fact that observations, such as those made by Libet, are not immune to such effects have been shown by Banks and Isham [4], who manipulated deceptive feedback on their movements to subjects performing a Libet-like task, thereby suggesting to them that their movement was performed later than it actually was. This led subjects to delay the time at which they reported “deciding” to perform the act. This fact would be impossible to explain if an illusion of making such a decision was purely a result of SMA activity occurring 500 ms prior to the action. However, it fits very well with the notion that these self-reports are interpretations that can be manipulated by circumstances.

One very simple and mundane explanation for the initial findings of SMA activity preceding the experience of free will may be that the experimental paradigms manipulated the ways subjects and patients perceived the situation. Patients who were stimulated on the SMA cortex may have perceived that their inclination to perform certain movements emanated from a neural area that is typically directly under executive control. Similarly, the fact that participants in the Libet experiment describe movements as caused by voluntary decisions may be caused by the fact that this system is initiating the movements. Based on these cues, these patients and participants honestly describe their reconstructive interpretations of the situation as consistent with the idea that their movements or urges to move were grounded in free will and free choices.

## Conclusions

So, what is the nature of the neural and behavioral system(s) that will produce the readiness potential in the SMA? Literature on the neural basis of volition, experiences from neurosurgical resections of the SMA, and some basic tenets and concepts of cognitive science may suggest something like the following: human non-stimulus-driven motor behavior is typically governed by reason and decision-making processes regarding ultimate goals. The proximal cognitive processes through which such decisions are made are often to a considerable extent, conscious. The execution of these decisions is however often monitored in a way that may not require unwavering conscious attention [18]. One example of a decision that may be the result of such conscious reasoning may be the decision to take part as a subject in a Libet-like experiment where you are required to flick your wrist at an unspecified moment.

The exact timing, precision and nature of a motor behavior that you have decided to perform, is in turn designed by neural systems for executive function that may involve prefrontal and parietal regions as well as part of the cingulum. Here the SMA functions as a gateway by which activity from these systems is channeled to the primary motor cortex and the corticospinal tract. Through this route, SMA activity above a certain threshold will produce motor movement.

Direct cortical stimulation of this gateway, i.e., SMA stimulation, on awake patients may shortcut this system producing a movement that is perceived as non-stimulus-driven (i.e., voluntary). Registration of activity in the SMA, i.e., through EEG recordings, may reveal a readiness potential when impulses for motor behavior are channeled through the SMA. When subjects are asked when they perceived or “decided” on the exact timing for such movements, they will report the time at which they perceived the activity induced by the SMA.

Resections of the SMA will remove the gateway by which non-stimulus-driven motor actions are initiated by activation of the primary motor cortex. However, the neural systems governing conscious, higher order decision-making will remain intact.

So, what does this model for SMA activity tell us about the question whether human behavior is completely determined and caused by lower order events, such as molecular processes in the brain? Well, it is notable that the model that would seem to be the most parsimonious explanation for the data reviewed above rests on an assumption of the existence of free conscious choices. However, this does of course not settle the debate from a purely philosophical point of view.

**Fig. 3:**
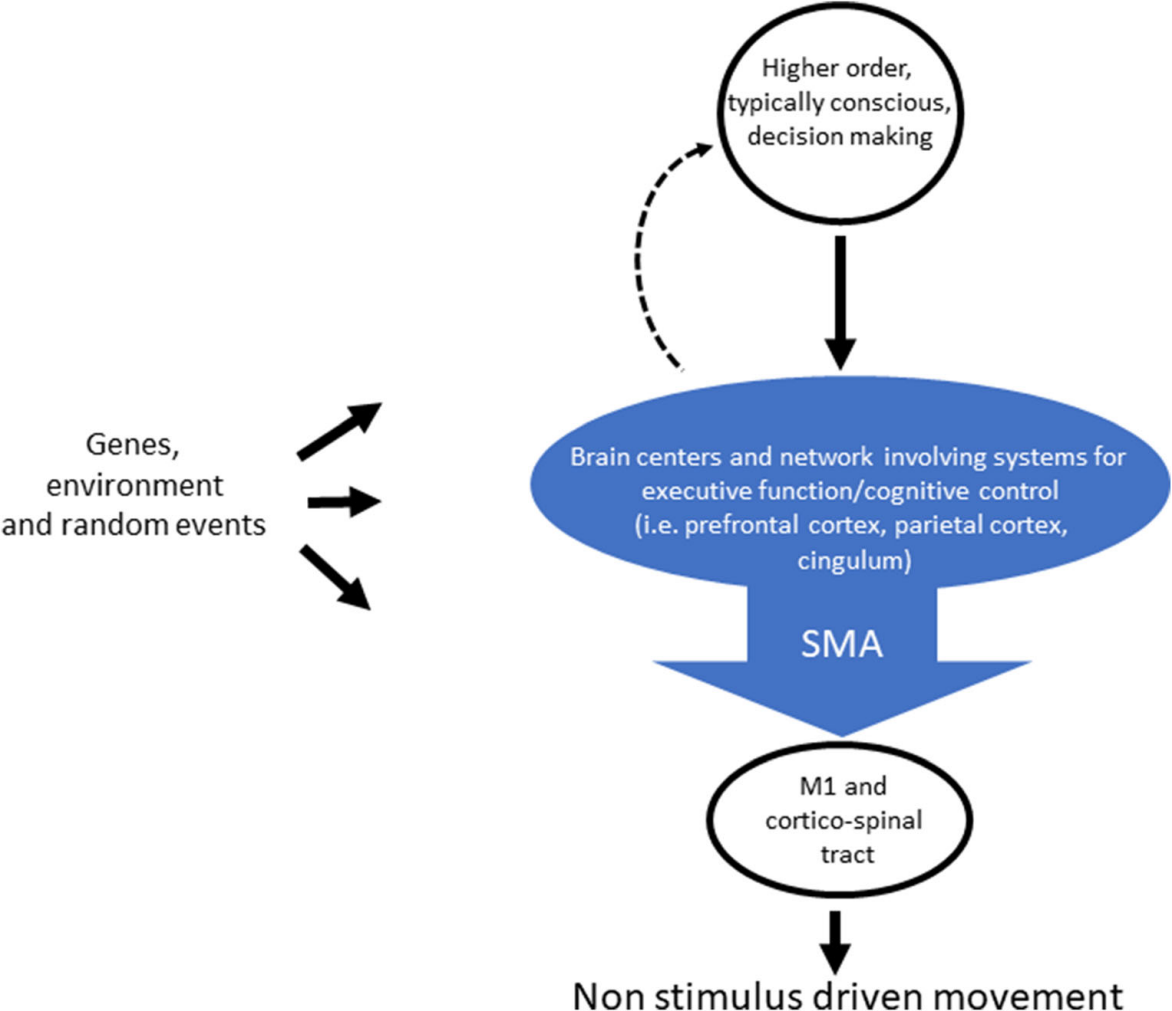
Models for simple non-stimulus-driven human motor actions that appear consistent with observations of patients after resections of the SMA. (M1 = primary motor cortex)

For many, the perhaps most tantalizing promise of the Libet experiment lay in the idea that the definitive answers to some of the most complex, abstract question in the history of human ideas could lie in the empirical study of a small piece of the brain. A part of the brain in which depth electrodes can be inserted, electrical stimulation applied, and which can even be removed using ultrasonic aspiration. The results of the neuroscientific study of, and the neurosurgical physical interactions with, the SMA during the latest half century have provided interesting and useful insights, some of which I have attempted to summarize above. However, the fundamental question if and how a belief in an almighty God, or a predetermined universe governed by laws of nature, can be reconciled with the idea of humans as being in possession of conscious free will cannot be definitely answered by models such as the one presented in Fig. 3.

## References

[1] Aristotle (1984). Nicomachean ethics J. Barnes (Ed.), The complete works of Aristotle: the revised Oxford translation, Vol.

[2] Augustine (1999) The confessions of Saint Augustine. Oak Harbor, WA: Logos Research Systems, Inc., (Original work published around 397) https://www.documentacatholicaomnia.eu/03d/0354-0430,_Augustinus,_Confessionum_Libri_Tredecim-Pusey_Transaltion,_EN.pdf

[3] Austin JT, Vancouver JB (1996). Goal constructs in psychology: structure, process, and content. Psychol Bull.

[4] Banks WP, Isham EA (2009). We infer rather than perceive the moment we decided to act. Psychol Sci.

[5] Bannur U, Rajshekhar V (2000). Post operative supplementary motor area syndrome: clinical features and outcome. Br J Neurosurg.

[6] Bartlett FC (1932). Remembering: a study in experimental and social psychology.

[7] Braem S, Bugg JM, Schmidt JR, Crump MJC, Weissman DH, Notebaert W, Egner T (2019). Measuring adaptive control in conflict tasks trends. Cogn Sci.

[8] Cashmore AR (2010). The Lucretian swerve: the biological basis of human behavior and the criminal justice system. Proc Natl Acad Sci U S A.

[9] Desmurget M, Reilly KT, Richard N, Szathmari A, Mottolese C, Sirigu A (2009) Movement intention after parietal cortex stimulation in humans Science.324(5928):811-3. 10.1126/science.1169896 10.1126/science.1169896 19423830

[10] Fried I, Haggard P, He BJ, Schurger A (2017). Volition and action in the human brain: processes, pathologies, and reasons. J Neurosci.

[11] Fried I, Katz A, McCarthy G, Sass KJ, Williamson P, Spencer SS, Spencer DD (1991). Functional organization of human supplementary motor cortex studied by electrical stimulation. J Neurosci.

[12] Fried I, Mukamel R, Kreiman G (2011). Internally generated preactivation of single neurons in human medial frontal cortex predicts volition. Neuron.

[13] Fourneret P, Jeannerod M (1998). Limited conscious monitoring of motor performance in normal subjects. Neuropsychologia.

[14] Gomes G (1998). The timing of conscious experience: a critical review and reinterpretation of Libet’s research. Conscious Cogn.

[15] Groff PS (2007). Islamic philosophy A-Z.

[16] Haggard P (2008). Human volition: towards a neuroscience of will. Nat Rev Neurosci.

[17] Heiferman DM, Ackerman PD, Hayward DM, Primeau MJ, Anderson DE, Prabhu VC. (2014) Bilateral supplementary motor area syndrome causing akinetic mutism following parasagittal meningioma resection Neurosci Disc, 2, article 7

[18] Horga G, Maia TV. Conscious and unconscious processes in cognitive control: a theoretical perspective and a novel empirical approach Front Hum Neurosci. 2012 Jul 4;6:199. 10.3389/fnhum.2012.00199.eCollection2012. 10.3389/fnhum.2012.00199 PMC3458455 23055959

[19] Hugdahl K, Asbjornsen A. (1994) Dikotisk lyssning med CV-stavelser Psykologiforlaget Manual: Hagersten

[20] Hugdahl K, Westerhausen R, Alho K, Medvedev S, Laine M, Hämäläinen H (2009). Attention and cognitive control: unfolding the dichotic listening story. Scand J Psychol.

[21] James W (1890). The principles of psychology.

[22] Johnson MK, Hashtroudi S, Lindsay DS (1993). Source monitoring. Psychol Bull.

[23] Johnson MK, Raye CL (1981). Reality monitoring. Psychoanal Rev.

[24] Kasasbeh AS, Yarbrough CK, Limbrick DD, Steger-May K, Leach JL, Mangano FT, Smyth MD (2012). Characterization of the supplementary motor area syndrome and seizure outcome after medial frontal lobe resections in pediatric epilepsy surgery. Neurosurgery.

[25] Kornhuber HH, Deecke L (1965). Hirnpotentialänderungen bei Willkürbewegungen und passiven Bewegungen des Menschen: Bereitschaftspotential und reafferente Potentiale. Pflüger’s Archiv für die gesamte Physiologie des Menschen und der Tiere.

[26] Krainik A, Lehéricy S, Duffau H, Vlaicu M, Poupon F, Capelle L, Cornu P, Clemenceau S, Sahel M, Valery CA, Boch AL, Mangin JF, Bihan DL, Marsault C (2001). Role of the supplementary motor area in motor deficit following medial frontal lobe surgery. Neurology.

[27] Laplane D, Talairach J, Meininger V, Bancaud J, Orgogozo JM (1977). Clinical consequences of corticectomies involving the supplementary motor area in man. J Neurol Sci.

[28] Lau HC, Rogers RD, Haggard P, Passingham RE (2004). Attention to intention. Science.

[29] Lau HC, Rogers RD, Passingham RE (2007). Manipulating the experienced onset of intention after action execution. J Cogn Neurosci.

[30] Lavazza A. (2016) Free will and neuroscience: from explaining freedom away to new ways of operationalizing and measuring it. Front Hum Neurosci 10: Article Number: 262. 10.3389/fnhum.2016.00262 10.3389/fnhum.2016.00262 PMC4887467 27313524

[31] Lezak M, Howieson D, Bigler E, Tranel D (2012) Neuropsychological assessment, fifth edn. Oxford University Press, New York

[32] Libet B, Sinnott-Armstrong W, Nadel L (2011). Do we have free will?. Conscious will and responsibility.

[33] Libet B, Gleason CA, Wright EW, Pearl DK (1983). Time of conscious intention to act in relation to onset of cerebral activity (readiness-potential): the unconscious initiation of a freely voluntary act. Brain.

[34] Lucretius (2008). On the nature of the universe.

[35] Maoz U, Yaffe G, Koch C, Mudrik L (2019). Neural precursors of decisions that matter—an ERP study of deliberate and arbitrary choice. Elife..

[36] Nann M, Cohen LG, Deecke L, Soekadar SR (2019). To jump or not to jump-the Bereitschaftspotential required to jump into 192-meter abyss. Sci Rep.

[37] Neisser U, Becklen R (1975). Selective looking: attending to visually specified events. Cogn Psychol.

[38] O’Connor T, Franklin C. (2020) Free will. The Stanford Encyclopedia of Philosophy (Fall 2020 Edition) Edward N Zalta (ed).

[39] Passingham RE, Bengtsson SL, Lau HC (2010). Medial frontal cortex: from self-generated action to reflection on one’s own performance. Trends Cogn Sci.

[40] Penfield W, Jasper H (1954) Epilepsy and the functional anatomy of the human brain. Boston: Little Brown, 1954.

[41] Penfield W, Welch K (1951). The supplementary motor area of the cerebral cortex: a clinical and experimental study. AMA Arch Neurol Psychiat.

[42] Pocket S, Purdy SC, Sinnott-Armstrong W, Nadel L (2011). Are voluntary movement initiated pre consciously? The relationship between readiness potentials, urges and decisions. Conscious will and responsibility.

[43] Racine E, Nguyen V, Saigle V, Dubljevic V (2017). Media Portrayal of a Landmark Neuroscience Experiment on Free Will. Sci Eng Ethics.

[44] Sapolsky RM. (2017) Behave. Penguin Press

[45] Scheperd J. (2015) Scientific challenges to free will and moral responsibility. Philosophy Compass, 10-197-207 10.1111/phc3.12200 PMC4479978 26146511

[46] Schurger A, Sitt JD, Dehaeney S (2012). An accumulator model for spontaneous neural activity prior to self-initiated movement. Proc Natl Acad Sci U S A.

[47] Seligman MEP, Railton P, Baumeister RF, Sripada C (2013). Navigating into the future or driven by the past. Perspect Psychol Sci.

[48] Sjöberg RL (1995). Child testimonies during an outbreak of witch hysteria: Sweden 1670-1671. J Child Psychol Psychiatry.

[49] Sjöberg RL (1997). False allegations of satanic abuse: case studies from the witch panic in Rättvik 1670-71. Eur Child Adolesc Psychiatry.

[50] Sjöberg RL, Lindholm T (2009). Children’s autobiographical reports about sexual abuse: a narrative review of the research literature. Nordic Journal of Psychiatry.

[51] Sjöberg RL, Stålnacke M, Andersson M, Eriksson J (2019). The supplementary motor area syndrome and cognitive control. Neuropsychologia.

[52] Soon CS, Brass M, Heinze H-J, Haynes J-D (2008). Unconscious determinants of free decisions in humans. Nat Neurosci.

[53] Stroop JR (1935). Studies of interference in serial verbal reactions. J Exp Psychol.

[54] Stålnacke M, Solowska K, Bergenheim T, Sjöberg RL (2020). Phenomenology of glioma resection in the dorsal medial frontal cortex. Acta Neurol Scand.

[55] Vassal M, Charroud C, Deverdun J, Le Bars E, Molino F, Bonnetblanc F, Boyer A, Dutta A, Herbet G, Moritz-Gasser S, Bonafé A, Duffau H, de Champfleur NM. (2017) Recovery of functional connectivity of the sensorimotor network after surgery for diffuse low-grade gliomas involving the supplementary motor area. J Neurosurg 126(4):1181-1190. 10.3171/2016.4.JNS152484 10.3171/2016.4.JNS152484 27315027

[56] Wallsten T, Kjellin L, Sjoberg RL (2008). The diagnostic accuracy of questions about past experiences of being mechanically restrained in a population of psychiatric patients. Memory..

[57] Wegner DM. (2002) The illusion of conscious will. Cambridge, MA: MIT

[58] Zentner J, Hufnagel A, Pechstein U, Wolf HK, Schramm J (1996). Functional results after resective procedures involving the supplementary motor area. J Neurosurg.

